# Adequately Iodized Salt Utilization and Associated Factors among Households in Tach Armachio District, Northwest Ethiopia: A Community-Based Cross-Sectional Study

**DOI:** 10.1155/2021/6630450

**Published:** 2021-04-16

**Authors:** Worku Mamo, Terefe Derso, Solomon Gedlu Nigatu

**Affiliations:** ^1^Carter Center Ethiopia, Addis Ababa, Ethiopia; ^2^Department of Human Nutrition, Institute of Public Health, College of Medicine and Health Sciences, University of Gondar, Gondar, Ethiopia; ^3^Department of Epidemiology and Biostatistics, Institute of Public Health, College of Medicine and Health Sciences, University of Gondar, Gondar, Ethiopia

## Abstract

**Background:**

For the synthesis of thyroid hormones, iodine is a crucial trace element. Iodine deficiency disorders affect all groups particularly: pregnant, young women and children. Iodine deficiency disorder has been recognized as a serious public health issue in Ethiopia. Therefore, this study planned to assess iodized salt utilization and associated factors at the household level.

**Methods:**

A community-based cross-sectional study was conducted from January 25 to February 24, 2019, in Tach Armachio district, Northwest Ethiopia. A single population proportion formula was used to calculate the sample size and a total of 555 households were sampled. A multistage sampling technique was conducted to select the household. An iodometric titration method was performed. A structured, pretested, and face-to-face interview questionnaire was used to collect data; then, it was entered in Epi Info 7 and exported to SPSS version 25 for analysis. Bivariable and multivariable analyses were done to identify predictor variables. A 95% confidence interval and adjusted odd ratio were reported. *P* values less than 0.05 were considered statistically significant in the multivariable analysis.

**Results:**

This study showed that iodized salt was adequately utilized by 61.1% (CI = 57%–65%) of households. Good knowledge of iodine deficiency disorder (AOR = 2.25, 95% CI = (1.44, 3.50)), keeping salt in the kitchen house away from fire (AOR = 5.09, 95% CI = (3.25, 7.98)), buying packed salt [AOR = 1.89, 95% CI = (1.12, 3.19)), keeping salt in a covered container (AOR = 2.18, 95% CI = (1.24, 3.81)), and exposing salt to sunlight (AOR = 0.39, 95% CI = (0.23, 0.65)) were significantly associated.

**Conclusion:**

In the district, adequately iodized salt utilization was low. Therefore, it is necessary to enforce the current law for merchants to sell iodized packed salt and teach the community how to handle it.

## 1. Introduction

Iodine is a crucial element for the synthesis of thyroid hormone (TH). This hormone is involved in the body's growth, development, reproduction, and metabolic process control [1]. Iodine deficiency disorders (IDDs) occur when the human body does not receive the recommended amount of iodine on a daily basis. IDDs can result in mental impairment, endemic goiter, hypothyroidism, reproductive failure, and dwarfism [2–4]. Salt iodization is the most cost-effective, widely used, and low-cost public health intervention for delivering the required amount of iodine to the general population [2, 4].

In 2017, less than 10 percent of the global population of the general population lived in countries categorized as iodine deficiency [5]. IDDs affect people of all ages, but young children, women of reproductive age, pregnant women, and lactating women are the most vulnerable [4]. IDDs remain a public health concern [6]. Previous studies in South Asia [7–10] and Sub-Saharan Africa [7, 11–14] have found that iodized salt is in short supply at the household (HH) level. In Ethiopia, pocket-area studies revealed that a large number of HHs used insufficiently iodized salt when compared to the universal standard [15–20]. However, according to the 2016 Ethiopian Demographic Health Survey, 89 percent of HHs used enough iodized salt [21].

The use of packed salt, not exposing salt to sunlight, storing salt in dry areas, shorter storage time, adding salt late after cooking, good knowledge of iodized salt and IDDs, and socioeconomic status are all factors that influence the availability of adequately iodized salt [7, 22–26]. Previously, a rapid test kit was used to assess iodine content qualitatively, but it was unreliable (low specificity) [27]. The iodometric titration method (IDTM) is the best and most preferred method. This method is reliable and is used to determine the amount of iodine in salt [28–30].

A good strategy for preventing IDDs is to ensure that the iodine content in salt is safe and sufficient at the HH level [29, 31]. It is necessary to pay attention to how such iodine salt should be stored. According to literatus because of the volatile of iodine, it will be lost due to high humidity and boiling during cooking. Even if the effect is small, light or dry heat causes iodine to be lost from the salt [32–34]. There is an inconsistency of findings in Ethiopia, and no studies have been done using the titration method in the current study area. Furthermore, the study area has a high prevalence of goiter, implying that iodized salt utilization will be poor [35, 36]. Therefore, this study used gold standard IDTM to assess the prevalence of adequately iodized salt usage and associated factors at the HH level in Tachar Amchio district, Northwest Ethiopia.

## 2. Materials and Methods

### 2.1. Study Design, Area, and Period

Tach Aramchio district is found in the Central Gondar Zone, Amhara region, Northwest Ethiopia. It is located 790 kilometers north of Addis Ababa and 63 kilometers south of Gondar Town. According to the 2011 Finance and Economic Development Bureau projections, the district had a total of 37,139 households and 162,354 people. The administration is divided into 32 kebeles (the smallest administrative unit of Ethiopia). The elevation ranges from 900 to 1200 meters above sea level. There is one district hospital, nine health centers, and 39 health posts. A community-based cross-sectional study was conducted from January 25 to February 24, 2019. The sample size was determined using a single population proportion formula. It accounts for a 33.2% prevalence [15], 95% confidence level (CI), 5% margin of error, and 1.5 design effect. It also included a 10% nonresponse rate, resulting in a sample size of 563 households. A multistage sampling procedure was used. Eight of the 32 kebeles were chosen by lottery in the first stage (one from the urban area and the other from the rural area). Second, an HH was chosen using a systematic sampling method, with every 15^th^ HH included in the study sample.

### 2.2. Data Collection Tools and Measurement

A structured, pretested, and face-to-face interview questionnaire was used to collect data. The questionnaire included sociodemographic questions, as well as questions about iodized salt and IDD knowledge (study participants who answered correctly 50% of nine questions about iodized salt and IDD knowledge were considered to have good knowledge, while those who scored below 50% were considered to have poor knowledge) [22], salt storage condition, and practice variables for handling. Four well-trained health extension workers collected the data.

### 2.3. Laboratory Procedures

To determine salt iodine content, IDTM was done. A 50 g mixed (homogenized) salt sample was taken from each systematically selected household, using a moisture-free, clean, and airtight plastic container. The sample was labeled and coded with the date of sampling, source of salt, and batch number. Each sample was analyzed in triplicate, and the average sample concentration was used to calculate the iodine concentration. The IDTM uses reagents like sulphuric acid, potassium iodate, and potassium iodide as principal reagents, standardized sodium thiosulphates (as titrant), and starch solution as an indicator. The titration results were converted to iodine concentrations and then classified based on their iodine content. The outcome variable, adequately iodized salt utilization measured as parts per million (ppm) < 15 was considered as inadequately iodined, while ≥15 ppm is considered as adequately iodized [30]. The test was carried out in an Ethiopian public health institution's laboratory.

### 2.4. Data Processing and Analysis

Data quality is maintained by ensuring that data is complete and consistent. It was entered into the Epidemiological Information (Epi Info) software version 7.1 and then transferred to Statistical Package for Social Sciences (SPSS) version 16 for analysis. The following calculations were made: mean, standard deviation, percentage, confidence interval, and odds ratio (OR). A principal component analysis was used to determine wealth status. Wealth is a latent variable which cannot measure directly using a single question. There were 15 questions dealing with productive assets, nonproductive assets, and household utilities. We used a factor reduction analysis. Our first step was that variables have been reclassified; we assign categories values with 0 and 1. Then, we check coefficients, KMO, Bartlett's test of sphericity, and eigenvalues. Finally, we rank the wealth index after ascending and created the quintiles as poor, medium, and rich. Bivariable logistic regression was used to see if there was a significant relationship between the dependent and independent variables. Variables with *P* values of 0.2 were included in the multivariable analysis during the bivariable analysis. A multivariable analysis was done to control a possible confounding effect of independent variables. In the multivariable analysis, a variable with a *P* value of less than 0.05 was considered statistically significant. The Hosmer and Lemeshow goodness of fit test for the model was also checked and it had a *P* value of 0.15. Multicollinearity among independent variables was checked using the Variance Inflation Factor (VIF) which indicates that there is no multicollinearity because all variables have VIF < 7 and tolerance greater than 0.1.

## 3. Result

A total of 555 households were involved in the study and eight HHs declined to participate which made the response rate 98.57%. The average age of respondents was 33.36 years with standard deviation ± 9.08. Almost half of the respondents 270 (48.6%) were between the ages of 34 and 44. The majority of the study participants (*n* = 459; 82.7%) were females by sex, and 317 (57.1%) were housewives by their occupation. Five hundred twenty-two (94.1%) of the respondents were Orthodox Christians.

Regarding marital status, 442 people (79.6%) were married. Nearly half of the study subjects' 263 participants (47.4%) could read and write. Three hundred and seventy-four (67.4%) of the HHS have less than five members. In family wealth status, 206 (37.1%) were classified as middle income (Table 1).

### 3.1. Knowledge of IDD, Storage Condition, and Utilization Practice of Iodized Salt at Households

The prevalence of adequately iodized salt is found in 61.1% (95% CI 57–65) of the study participants, which is >15 ppm. The median iodine content of the sampled salt was 18.03 ppm, with an interquartile range of 11.66 ppm to 28.34 ppm. The amount of salt in the 15–40 ppm range was 42.2%.

Respondents with good knowledge of IDDs and iodized salt were 312 (56.2%). The majority of study participants (*n* = 417; 75.1%) walked less than an hour to buy salt, and two-thirds (77.5%) did so once a week. Households that purchased it from a retail store and in packed form numbered 312 (56.2%) and 432 (77.8%), respectively.

Almost all of the 545 (98.2%) HHs stored their salt in a dry place. Of these, 467 (84.1%) kept their salt away from sunlight (Table 2).

### 3.2. Associated Factors of Adequate Iodized Salt

When HH heads with good knowledge of IDD were compared to those with poor knowledge, they were 2.25 times more likely to have adequate iodized salt (AOR = 2.25, 95% CI: 1.44–3.50). Respondents who place their salt away from the fire in the kitchen were 5.09 times more likely to have adequately iodized salt compared to those placed near to the fire (AOR = 5.09, 95% CI: 3.25–7.98).

The odds ratio of using adequate iodized salt is 89% higher among those who buy packed salt compared to unpacked salt (AOR = 1.89, 95% CI: 1.12–3.19). Study participants who stored their salt in a covered container were 2.18 times more likely to have adequately iodized salt as compared to those who did not (AOR = 2.18, 95% CI: 1.24–3.81). The odds of adequately iodized salt usage decreased by 61% among those who exposed their salt to sunlight (AOR = 0.39, 95% CI: 0.23–0.65) (Table 3).

## 4. Discussion

According to the WHO and International Council for Control of Iodine Deficiency Disorders (ICCIDD) standard, the elimination of IDD will be possible if more than 90% of the households utilized adequately iodized salt [37]. The current study revealed that 61.1% of households had adequately iodized salt (>15 ppm), falling short of the universal salt iodization (USI) target of 90% coverage. This result was lower than those found in studies from Saudi [37], Bangladesh [26], Nepal [38], and Dessie town, Ethiopia [19]. It was, however, higher than the studies of Morocco [39], Sudan [16], and four studies from Ethiopia [17–19, 21]. It is in line with research conducted in Ethiopia's Asella town [40], Dera district [41], and Wondo Genet town [20]. The possible reason for this difference includes the use of IDTM to measure iodine content in the current study, difference in the study population, governments' commitment to enforcing legislation, and regulations on the iodization of salt, distribution, and retail. Ethiopian Food and Drug Authority issued a directive in 2011 to prohibit the distribution and retail of noniodized salt. If the directive is violated, the trade license will be temporarily suspended for one to six months, and in the worst case, the license will be permanently revoked [42].

In this study, the odds of having adequately iodized salt in a household was higher among participants who had good knowledge of IDD and iodized salt utilization than poor knowledge. This finding is supported by the studies in Ghana [23], Bensa woreda [43], Laelay Maychew [23], and Dabat [15], Ethiopia. These could be respondents (households) who are already aware and can easily put their knowledge into practice by purchasing iodized salt.

Households who keep their salt away from the fire in the kitchen were more likely to have adequate iodized salt compared to those that kept their salt near the fire. This could result from heat-induced alteration or depletion of iodine as it is volatile by nature. As a result, the respondents who had placed salt away from the fire and protected it from the heat in the kitchen had adequately iodized salt [44].

HHs who used packaged salt were more likely to have adequate iodized salt than those who used bulk salt. This finding is supported by the studies done in Bensa woreda [43], Gondar town [22], and Dabat [23]. This may have been packing material that protected the salt from exposure to sunlight and kept it from moisture.

Households that used salt stored in covered containers were positively associated with adequately iodized salt. Salt placed in a covered container was 1.92 times more likely to have adequately iodized salt than salt stored in open containers, and this study is consistent with the studies in Dera and Lalo Assabi district, Ethiopia [41, 45]. The iodine content of the salt may be maintained or retained in the covered container. Also, a covered container prevents salt from being exposed to light and keeps it dry.

At the HH level, exposing salt to sunlight was found to be inversely related to adequate iodized salt utilization. This finding is consistent with research from Wolita [18], Goba town [46], Dera district [45], and Gondar town [22], Ethiopia. This could be the result of sunlight (heat) slowly evaporating the iodine content (volatile nature of iodine content) from the HH's use of iodized salt.

### 4.1. Limitations of the Study

The study's limitation was that the use of iodized salt in retail stores was not measured, and the labeling time was not checked. The impact of clusters between urban and rural areas is not being considered. In responding to handling practices, this study was not free of social desirability bias.

## 5. Conclusion and Recommendation

In the district, the proportion of people who use enough iodized salt in their homes is still low. The location of storage, the type of salt purchased, whether the salt was stored in a covered container, and whether the salt was exposed to sunlight have all been identified as significant predictors. As a result, it is necessary to enforce the existing law prohibiting traders from selling unpacked iodized salt and educate the public about how to use iodized salt in the kitchen.

## Figures and Tables

**Table 1:** Sociodemographic and economic characteristics of respondents, Tach Armachiho district, Northwest Ethiopia 2019 (*N* = 555).

Variables	Category's	Frequency (*N*)	Percentage
Sex	Male	96	17.3
	Female	459	82.7

Age in year	17–29	213	38.4
	30–44	270	48.6
	≥45	72	13

Residence	Urban	300	54.1
	Rural	255	45.9

Religion	Orthodox	522	94.1
	Muslim	33	5.9

Marital status	Single	50	9
	Married	442	79.6
	Divorced	52	9.4
	Widowed	11	2

Educational status of study participant	Unable to read and write	108	19.4
	Read and write	263	47.4
	Primary	103	18.6
	Secondary and above	81	14.6

Educational status of head of household	Unable to read and write	43	7.8
	Read and write	301	54.2
	Primary	100	18
	Secondary and above	111	20

Occupation of study participant	Government employee	28	5.1
	Merchant	67	12.1
	Housewife	317	57.1
	Self-employed	124	22.3
	Others^*∗*^	19	3.4

Family size in number	<5	374	67.4
	≥5	181	32.6

Wealth status	Poor	177	31.9
	Medium	206	37.1
	Rich	172	31.0

^*∗*^Students, daily laborer, childhood and family members.

**Table 2:** Storage condition and utilization practice of iodized salt at household level in Tach Armachiho district, Northwest Ethiopia 2019 (*N* = 555).

Variables	Category's	Frequency (*N*)	Percentage
Used of ID salt cooking food	Yes	463	83.4
	No	92	16.6

Time salt added to cook in the last 24 hours	Early at the beginning of cooking	14	2.5
	Late in the middle of cooking	61	11.0
	At the end of cooking	354	63.8
	Right after cooking	126	22.7

Storage place in kitchen house	Near to the fire	149	26.8
	Away from the fire	406	73.2

Used covered container	Yes	483	87
	No	72	13

Storage container	Dry place	545	98.2
	Moisture place	10	1.8

Exposed to sunlight	Yes	88	15.9
	No	467	84.1

Type of salt	Packed	432	77.8
	Bulk	123	22.2

Where did you buy salt?	Retail shop	312	56.2
	Open market	213	38.4
	Wholesale	30	5.4

Distance travel to buy salt	≤1 hr walking on foot	417	75.1
	>1 hr walking on foot	138	24.9

Frequency of buying salt	Once a week	74	13.3
	Once a month	430	77.5
	Once in more than a month	51	9.2

Wash salts before consumption	Yes	13	2.3
	No	542	97.7

Use ID salt to preserve food	Yes	180	32.4
	No	176	31.7
	I do not remember	199	35.9

Salt iodine content ppm	1–14.99	216	38.9
	15–40	234	42.2
	>40	105	18.9

**Table 3:** Factors associated with adequate iodized salt utilization in Tach Armachiho district, Northwest Ethiopia 2019 (*N* = 555).

Variables	Categories'	Adequate iodized salt utilization		Odds ratios		*P* value
		Yes (≥15 ppm)	No (<15 ppm)	COR (95%CI)	AOR (95% CI)	
Family size	<5 members	241	133	1.54 (1.07–2.20)	1.37 (0.88–2.12)	0.16
	≥5 members	98	83	1	1	

Place of residence	Urban	193	107	1.35 (0.96–1.89)	0.63 (0.37–1.08)	0.90
	Rural	146	109	1	1	

Educational status of study participant	Not educated	59	49	0.67 (0.37–1.21)	1.36 (0.63–2.93)	0.43
	Read and write	158	105	0.84 (0.50–1.41)	1.23 (0.65–2.30)	0.53
	Primary (1–8)	70	33	1.18 (0.64–2.18)	1.55 (0.78–3.11)	0.21
	Secondary and above	52	29	1	1	

Knowledge of IDD and iodized salt utilization	Good	215	97	2.13(1.50–3.01)	2.25(1.44–3.50)^*∗∗*^	0.001
	Poor	124	119	1	1	

Storage place in kitchen house	Near to the fire	54	95	1	1	
	Away from the fire	285	121	4.14 (2.78–6.15)	5.09 (3.25–7.98)^*∗∗*^	0.001

Where did you buy salt?	Retail shop	209	103	2.32 (1.09–4.93)	1.03 (0.42–2.51)	0.94
	Open market	116	97	1.37 (0.64–2.94)	0.68 (0.28–1.68)	0.71
	Wholesale	14	16	1	1	

Distance travel to buy salt	<or = 1 hr waking	263	154	1.39 (0.94–2.06)	0.87 (0.49–1.53)	0.62
	>1 hr waking	76	72	1	1	

Type of salt	Packed	281	151	2.09 (1.39–3.13)	1.89 (1.12–3.19)^*∗*^	0.02
	Bulk	58	65	1	1	

Used covered container	Yes	305	178	1.92 (1.16–3.15)	2.18 (1.24–3.81)^*∗*^	0.01
	No	34	38	1	1	

Exposed to sun light	Yes	40	48	0.46 (0.29–0.74)	0.39 (0.23–0.65)^*∗∗*^	0.001
	No	299	168	1	1	

COR: crude odds ratio, CI: confidence interval, AOR: adjusted OR. ^*∗*^Significant at *P* < 0.05 and ^*∗∗*^significant at *P* < 0.01.

## Data Availability

The data are available upon request made to the corresponding author via email.

## Abbreviations

AOR: Adjusted odds ratio
CRO: Crude odds ratio
EDHS: Ethiopian Demographic and Health Survey
HHs: Households
IDDs: Iodine deficiency disorders
IDTM: Iodometric titration method
ppm: Parts per million
SPSS: Statistical Package for Social Sciences
USI: Universal salt iodization
WHO: World Health Organization.
